# Reclaiming AI as a Theoretical Tool for Cognitive Science

**DOI:** 10.1007/s42113-024-00217-5

**Published:** 2024-09-27

**Authors:** Iris van Rooij, Olivia Guest, Federico Adolfi, Ronald de Haan, Antonina Kolokolova, Patricia Rich

**Affiliations:** 1 Donders Institute for Brain, Cognition, and Behaviour, Radboud University, Nijmegen, The Netherlands; 2 Department of Cognitive Science and Artificial Intelligence, Radboud University, Nijmegen, The Netherlands; 3 Department of Linguistics, Cognitive Science, and Semiotics & Interacting Minds Centre, Aarhus University, Aarhus, Denmark; 4 Ernst Strüngmann Institute for Neuroscience in Cooperation with Max-Planck Society, Frankfurt, Germany; 5 School of Psychological Science, University of Bristol, Bristol, UK; 6 Institute for Logic, Language and Computation (ILLC), University of Amsterdam, Amsterdam, The Netherlands; 7 Department of Computer Science, Memorial University of Newfoundland, Newfoundland, Canada; 8 Department of Philosophy, University of Bayreuth, Bayreuth, Germany

**Keywords:** Artificial Intelligence (AI), Theory, Explanation, Engineering, Cognitive science, Computational complexity

## Abstract

The *idea* that human cognition is, or can be understood as, a form of computation is a useful conceptual tool for cognitive science. It was a foundational assumption during the birth of cognitive science as a multidisciplinary field, with Artificial Intelligence (AI) as one of its contributing fields. One conception of AI in this context is as a provider of computational tools (frameworks, concepts, formalisms, models, proofs, simulations, etc.) that support theory building in cognitive science. The contemporary field of AI, however, has taken the *theoretical* possibility of explaining human cognition as a form of computation to imply the *practical feasibility* of realising human(-like or -level) cognition in factual computational systems, and the field frames this realisation as a short-term inevitability. Yet, as we formally prove herein, creating systems with human(-like or -level) cognition is intrinsically computationally intractable. This means that any factual AI systems created in the short-run are at best decoys. When we think these systems capture something deep about ourselves and our thinking, we induce distorted and impoverished images of ourselves and our cognition. In other words, AI in current practice is deteriorating our theoretical understanding of cognition rather than advancing and enhancing it. The situation could be remediated by releasing the grip of the currently dominant view on AI and by returning to the idea of AI as a theoretical tool for cognitive science. In reclaiming this older idea of AI, however, it is important not to repeat conceptual mistakes of the past (and present) that brought us to where we are today.

## Introduction

The term ‘Artificial Intelligence’ (AI) means many things to many people (see Table 1 for different types of meanings). Sometimes, the term ‘AI’ is used to refer to the *idea* that intelligence can be recreated in artificial systems (Russell & Norvig, 2010). Other times, it refers to an artificial *system* believed to implement some form of intelligence (i.e., ‘an AI’). Some claim that such an AI can only implement domain-specific intelligence. An example could be an AI playing chess, where there is a fixed problem space defined by a fixed board, a limited number of pieces, and a small set of well-defined rules. Such a domain-specific AI can play chess, but cannot do the dishes or do medical diagnosis. Others believe that domain-general AIs—also known as artificial general intelligence (AGI)—can exist (Bubeck et al., 2023; cf. Birhane, 2021.) This domain generality can be seen as a key property of human intelligence, so that AGI would be human-level AI, able to incorporate arbitrary beliefs to solve arbitrary new problems. A person can not only play a game of chess, but reason about why their opponent has angrily left the room, and later draw on that event when writing a novel. In the history of both cognitive science and AI, it is generally understood that this domain generality is what makes human-like cognition so hard to explain, model, and replicate computationally (Fodor, 2000; Pylyshyn, 1987; van Rooij et al., 2019).^1^

The term ‘AI’ is also used to refer to the research and/or engineering *field* pursuing the creation of AI systems based on the idea that doing so is possible and desirable. AI as a research field is historically diverse (see Table 1), including AI-as-engineering approaches that focus on different domains of application and draw on different modeling and engineering frameworks, including not only the currently popular machine learning approach, but also approaches involving Bayesian Networks, decision support systems, and robotics. Hence, the ambition to build AGI is a subfield of the broader field of AI.

Among the more troublesome meanings of ‘AI’, perhaps, is as the ideology that it is desirable to replace humans (or, specifically women) by artificial systems (Erscoi et al., 2023) and, generally, ‘AI’ as a way to advance capitalist, kyriarchal^2^, authoritarian and/or white supremacist goals (Birhane & Guest, 2021; Crawford, 2021; Erscoi et al., 2023; Gebru & Torres, 2024; Kalluri, 2020; Spanton & Guest, 2022; Stark & Hutson, 2022; McQuillan, 2022). Contemporary guises of ‘AI’ as idea, system, or field are also sometimes known under the label ‘Machine Learning’ (ML), and a currently dominant view of AI advocates machine learning methods not just as a practical method for generating domain-specific artificial systems, but also as a royal road to AGI (Bubeck et al., 2023; DeepMind, 2023; OpenAI, 2023). Later in the paper, when we refer to AI-as-engineering, we specifically mean the project of trying to create an AGI system through a machine learning approach.

One meaning of ‘AI’ that seems often forgotten these days is one that played a crucial role in the birth of cognitive science^3^ as an interdiscipline in the 1970s and ’80s. Back then, the term ‘AI’ was also used to refer to the aim of using computational tools to develop theories of natural cognition. As Simon (1983, p. 27) put it 40 years ago,

Artificial Intelligence has two goals. First, AI is directed toward getting computers to be smart and do smart things so that human beings don’t have to do them. And second, AI (sometimes called cognitive simulation, or information processing psychology) is also directed at using computers to simulate human beings, so that we can find out how humans work.

This view of ‘AI’ as a research field overlapping with psychology sees computational AI systems as theoretical tools: ‘Many early AI researchers were concerned with using computers to model the nature of people’s thinking’ (Langley, 2006).^4^

**Table 1:** A non-comprehensive list of different (not mutually exclusive) meanings of the word AI, including AI as idea, AI as a type of system, AI as a field of study, and AI as institution(al unit)

Type	Description	Label
Idea	Intelligence can be recreated in artificial systems	AI-as-engineering
	Cognition is, or can be understood as, a form of computation	AI-as-psychology (a.k.a. computationalism)
	Humans can be replaced by artificial systems	AI-as-ideology
	The label ‘AI’ helps to sell technologies and gain funding	AI-as-marketing
System	A system believed to implement (simulate) a form of cognition	Cognitive system (model)
	A system believed to perform (solve) domain-specific cognitive tasks (problems)	Narrow AI
	A system believed to perform (solve) domain-general cognitive tasks (problems; what some may also call AGI)	General AI or AGI
	A system believed to realize human-level cognition (what some may also call AGI)	Human-level AI
Field	A (sub)field pursuing the creation of domain-specific AI systems	E.g. Bayesian Networks, Decision Support Systems, Machine Learning, Robotics
	A (sub)field pursuing the creation of AGI	AGI
	A (sub)field using AI as an idea to build theories	E.g., (computational) cognitive science, cognitive simulation, weak AI
	A field defined by a collection of fields that each are considered to be an AI subfield	The field of AI broadly construed
History	A history of practices reflecting different ideas of AI, resulting in the pursuit of different kinds of AI systems, and different kinds of AI-as-field concepts	Named to match practices, e.g. ML-AI, neuroAI, etc
Unit	An organisational or institutional unit going under the label AI	Named to match type of units, e.g. AI research group, AI department, AI centre, AI network

**Fig. 1:**
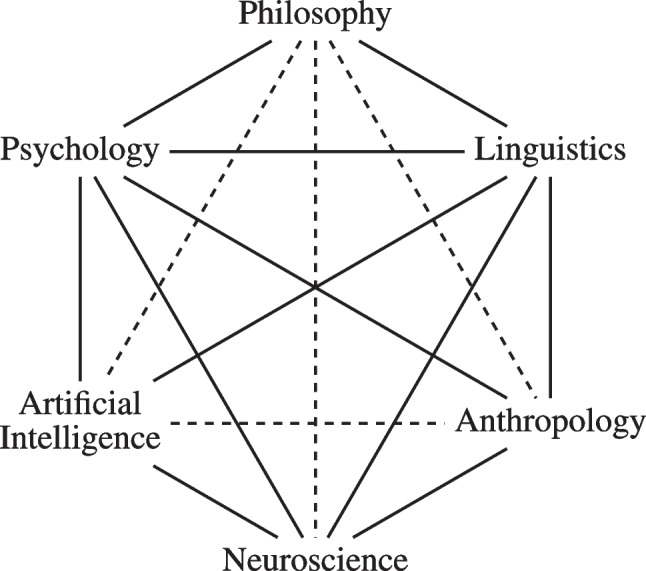
A visual depiction of the connections between the cognitive sciences. Solid lines denote stronger interdisciplinary ties, and dashed lines denote weaker ones. This figure is derived from the original put forth by the Sloan Foundation in 1978 and reproduced from Fig. 4 in Pléh and Gurova (2013). Different versions of it over time have used ‘Artificial intelligence’ (as above) instead of ‘Computer Science’ and vice versa (cf. Miller, 2003)

Accordingly, AI is one of the cognitive sciences (Fig. 1), and for decades, there was a close dialogue between the fields of AI and cognitive psychology (Forbus, 2010; Gentner, 2010, 2019; Miller, 2003). This is furthermore illustrated by the use of ‘cognitive simulation’ and ‘information processing psychology’ as alternative labels for ‘AI,’ favoured by Simon and associates.^5^ It is also illustrated by publications of cognitive psychological modelling research in Artificial Intelligence journals up to the ’90 s (Anderson, 1984; Cooper, et al., 1996; Thagard et al., 1990) and early 2000 s (Thagard, 2007), with still some notable exceptions these days. At the turn of the millennium, however, these productive ties between AI and psychology became severed:

the past 20 years have seen an increasing shift in AI research away from concerns with modelling human cognition and a decreasing familiarity with results from psychology. What began as a healthy balance [...] has gradually become a one-sided community that believes AI and psychology have little to offer each other. (Langley, 2006, p. 2)

AI qua information processing psychology was built on the idea that human cognition is, or can be scientifically understood as, a form of computation; this view is also known as *(minimal) computationalism*^6^ (cf. Chalmers, 2011; Dietrich, 1994; Milkowski, 2013). Computationalism was seen to provide useful conceptual tools for cognitive science (Boden, 1988, 2008; Hardcastle, 1995, 1996; Johnson-Laird, 1988) as it affords explicit specification of hypothesized cognitive processes and reasoning through the implications of such hypotheses (e.g. with mathematical proofs or computer simulations). However, present-day AI hype and the popularity of AI as technology (Meredith Whittaker, Edward Ongweso Jr., & Sarah Myers West in Denvir et al., 2023; Larson, 2021; Timnit Gebru in Marx & Wickham, 2023; van Rooij, 2023) and AI as a money-maker (Crawford, 2021) seems to leave little room for AI as a theoretical tool for cognitive science. The reason is that BigTech currently dominates the narrative, with a focus on technological progress and impressive machine learning applications. In practice, we observe that this leads to other, more theoretical perspectives on AI being overlooked or even forgotten. Worse still, the products of present-day AI-as-engineering are sometimes believed to instantiate (parts of) minds. Besides the various psychological, social, cultural and political problems posed by this confusion (Bender, et al., 2021; Birhane & van Dijk, 2020; Erscoi et al., 2023; J. Hughes, 2021; Larson, 2021; Thrall et al., 2018; Vallor, 2015; van der Gun & Guest, 2023; Wood, 1987), here we wish to focus on how this practice creates distorted and impoverished views of ourselves and deteriorates our theoretical understanding of cognition, rather than advancing and enhancing it.

In this paper, we wish to remedy the above situation in two steps. First, we set out to release the grip of the currently dominant view on AI (viz., AI-as-engineering aiming at a human-level AI system, Table 1). This practice has taken the *theoretical* possibility of explaining human cognition as a form of computation to imply the *practical feasibility* of realising human(-like or -level) cognition in factual computational systems, and it is framing this realisation as a short-term inevitability. In this paper, we undercut these views and claims by presenting a mathematical proof of inherent intractability (formally, NP-hardness) of the task that these AI engineers set themselves. This intractability implies that any factual AI system created in the short-run (say, within the next few decades or so) is so astronomically unlikely to be anything like a human mind, or even a coherent capacity that is part of that mind, that claims of ‘inevitability’ of AGI within the foreseeable future are revealed to be false and misleading. We realize that this implication may appear counterintuitive given everyday experiences and interactions with currently impressive AI systems, but we will explain why it is not. As we will carefully unpack later in the paper, it is a mistake to assume that AI systems’ performance is either currently human-level, or will simply continue to improve and the systems will soon constitute human-level A(G)I. The problem is that—in line with our intractability result—the performance cannot scale up (see Box 1).

Second, we propose a way to return to the idea of AI as a theoretical tool without falling in the trap of confusing our maps for the territory. The return must be such that we do not retrace the trajectory that led us where we are today. To halt the rerun of history, we think it is vital that the idea of cognition as computation—and therefore the in-principle possibility of realising and/or explaining cognition as a form of computation—is not mistaken for the practical feasibility of replicating human minds in machines (which we prove is not feasible). The reader may think that this is a contradiction, i.e. that then computationalism is theoretically inert. But that is mistaken. Computationalism can provide both explanatory challenges for and computational constraints on cognitive explanations—using formal, conceptual, and mathematical analysis—and hence is theoretically informative.

### Overview

*Flying pigs are also possible in principle; possible in principle bakes no bread.*

— Jerry Fodor (2005, p. 27)

The remainder of this paper will be an argument in ‘two acts’. In **ACT 1: Releasing the Grip**, we present a formalisation of the currently dominant approach to AI-as-engineering that claims that AGI is both inevitable and around the corner. We do this by introducing a thought experiment in which a fictive AI engineer, Dr. Ingenia, tries to construct an AGI under *ideal* conditions.^7^ For instance, Dr. Ingenia has perfect data, sampled from the true distribution, and they also have access to any conceivable ML method—including presently popular ‘deep learning’ based on artificial neural networks (ANNs) and any possible future methods—to train an algorithm (“an AI”). We then present a formal proof that the problem that Dr. Ingenia sets out to solve is intractable (formally, NP-hard; i.e. possible in principle but provably infeasible; see Section “Ingenia Theorem”). We also unpack how and why our proof is reconcilable with the apparent success of AI-as-engineering and show that the approach is a theoretical dead-end for cognitive science. In “**ACT 2: Reclaiming the AI Vertex**”, we explain how the original enthusiasm for using computers to understand the mind reflected many genuine benefits of AI for cognitive science, but also a fatal mistake. We conclude with ways in which ‘AI’ can be reclaimed for theory-building in cognitive science without falling into historical and present-day traps.

## ACT 1: Releasing the Grip

*There is no doubt that [AI] is currently in the process of rapid hill-climbing. Every year, states of the art across many [AI] tasks are being improved[, but] the question is whether the hill we are climbing so rapidly is the right hill.*

— Emily M. Bender and Alexander Koller (2020, p. 5191)

At present, the field of AI is in the grip of a dominant paradigm that pushes a narrative that AI technology is so massively successful that, if we keep progressing at our current pace, then AGI will inevitably arrive in the near future. Some multi-million AI companies have even gone so far as to claim that we are not only climbing a hill towards AGI, but even on the verge of creating AGI, whether we want to or not, if we proceed with current ML approaches to AI-as-engineering. These kinds of claims may be hard to ignore or counter if anything that is possible *in principle* also seems possible *in practice*. However, not everything that is possible in principle *is *possible in practice.

In this first *Act*, we reveal why claims of the inevitability of AGI walk on the quicksand of computational intractability. The main character on the stage is Dr. Ingenia, a fictive AI engineer, who is pursuing the kind of ML approach claimed to inevitably lead to AGI. By studying the engineering task that they have set themselves through a formal, mathematical lens, we are able to construct a proof of intractability. We then draw out its implications.

### Formalising AI-by-Engineering

*Give a rigorous, computationally detailed and plausible account of how learning can be done.* Translation: *Rigorous:* theorems, please.

— Dana Angluin (1992, p. 351)

**Fig. 2:**
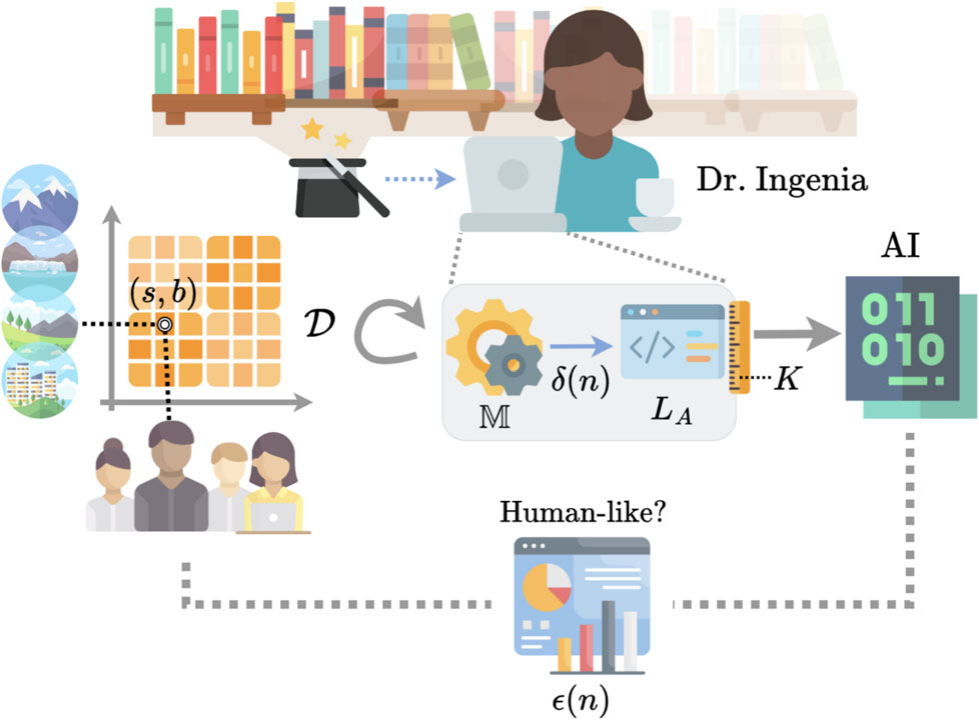
A visual illustration of the hypothetical scenario and its formalisation: Dr. Ingenia has access (magically and at no cost) to any machine learning method $\mathbb {M}$, present or future, and by repeatedly sampling data *D* from the distribution $\mathcal {D}$, they can use whatever $\mathbb {M}$ they like to create a program $L_A$ that when implemented and run generates behaviours $b = A(s)$ when prompted by different situations *s*. The goal is to generate with non-negligible probability $\delta (n)$ an algorithm *A* that behaves (approximately) human-like, in the sense that *A* is non-negligibly ($\epsilon (n)$) better than chance at picking behaviours that are possible for *s* in $\mathcal {D}$. Here, *n* is a measure of the situation complexity, i.e. the maximum length of strings (|*s*|) needed to encode the relevant information in the situations *s*

Imagine a fictive engineer, called Dr. Ingenia, who is trying to build human-like or -level AI (or AGI)^8^ using the currently dominant approach of ‘machine learning’ (in short, ‘ML’) under highly idealised and optimal conditions (Fig. 2). Dr. Ingenia is able to repeatedly sample from a distribution $\mathcal {D}$. Here, $\mathcal {D}$ captures possible behaviours *b* that humans may display in different possible situations *s*. Dr. Ingenia can use the information gathered by repeatedly sampling situation-behaviour pairs from $\mathcal {D}$ to try to build (or ‘train’) an algorithm. Their goal is to make an algorithm that behaves (approximately) like $\mathcal {D}$. Informally, we can characterise the problem that Dr. Ingenia sets themselves, as follows.^9^

AI-by-Learning (informal)

*Given*: A way of sampling from a distribution $\mathcal {D}$.

*Task*: Find an algorithm *A* (i.e., ‘an AI’) that, when run for different possible situations as input, outputs behaviours that are human-*like* (i.e., approximately like $\mathcal {D}$ for some meaning of ‘approximate’).

In our hypothetical scenario, we will be granting computationalism, i.e. we will not be challenging the assumption that cognition is a form of computation. Hence, Dr. Ingenia can safely assume that $\mathcal {D}$ is generated by a computational process, i.e. either by the computational processes that define a single individual’s cognition or the cognition of a finite collection of human beings. This also means that there exists an algorithm *A* that can approximate the distribution, namely, the algorithm that generates $\mathcal {D}$. But there may also be many more algorithms that deviate in some way from human cognition but whose behaviour is still sufficiently human-like.

Because our aim will be to assess the *intrinsic* hardness of Dr. Ingenia’s machine learning problem—independent of extraneous factors—we will be granting them several idealised conditions. For instance, in our hypothetical scenario, Dr. Ingenia will have access to *perfect* data for training their AI system. The data have no measurement error, nor are the data contaminated by irrelevant details or sampling bias or any other distortion on the data that make it indirect or imperfect. Note that the real-world situation is far removed from this idealized scenario. In contemporary ML, the predominant protocol is to train ML algorithms on data which is typically decontextualized and scraped from the internet (Birhane, et al., 2021, 2023; Lee et al., 2023; Liesenfeld et al., 2023, July). Moreover, real AI engineers do not have perfect knowledge of which cognizer contributed which data point, of which exact situation induced which behaviour, or of how behaviour is to be parsed, (cf. ‘the segmentation problem’; Adolfi et al., 2022; Adolfi et al., 2023) or interpreted (cf. ‘theory-ladeness of data’; Andrews, 2023; Guest & Martin, 2021; Hughes, 1997.)

By granting Dr. Ingenia these highly idealised conditions that simplify and abstract away from real-world complications, we can formally derive a reliable *lower*-bound on the real-world complexity of constructing human-like AI from human data. To do so, we need to make the problem AI-by-Learning formally precise such that it becomes amenable to computational complexity analysis (Arora & Barak, 2009; Garey & Johnson, 1979; van Rooij et al., 2019). We will do this next.

We assume that Dr. Ingenia expresses candidate algorithms *A* using a specification language, $\mathcal {L}_\mathcal {A}$. Any particular algorithm $A \in \mathcal {A}$ can be described with a program $L_A \in \mathcal {L}_\mathcal {A}$. The specification language $\mathcal {L}_\mathcal {A}$ can be thought of as a programming language with the constraint that it specifies only those algorithms in a class ${\mathcal {A}}$ that the engineer assumes is suitable for designing human(-like or -level) AI. For instance, $\mathcal {A}$ could be the class of artificial neural networks (ANNs) or any other class of algorithms that Dr. Ingenia deems sufficient to approximate human cognition.

We will add some minimal constraints on $\mathcal {L}_\mathcal {A}$. We exclude classes of trivial algorithms that have no chance of capturing human-like or -level cognition. Specifically, we impose the constraint that $\mathcal {L}_\mathcal {A}$ can *minimally* express feedforward neural networks, logical circuits, or finite state machine-equivalent class of algorithms. Scientifically, we think that a stronger assumption would be warranted, namely that $\mathcal {L}_\mathcal {A}$ is in principle expressive enough to be Turing complete, e.g. $\mathcal {L}_\mathcal {A}$ can express Turing machines (Turing, 1950) or otherwise Turing-equivalent algorithms, including certain (highly idealised) recurrent neural networks (Siegelmann & Sontag, 1992; Pérez et al., 2019; Weiss et al., 2018). This stronger assumption would ensure that $\mathcal {L}_\mathcal {A}$ is expressive enough to computationally capture human cognition. It would be a reasonable assumption because cognition is generally assumed to have two properties, known as *productivity* (i.e. people can in principle generate infinitely many distinct thoughts, sentences, images, etc., Fodor, 2005; Fodor & Pylyshyn, 1988) and *Turing-completeness* or*-equivalence* (the ability to compute any computable function, aided by pen and paper, in principle; Turing, 1950; Wells, 1998) We nonetheless work with the more modest assumption.

We will furthermore allow (but not impose) the assumption that all *A* expressible by $\mathcal {L}_\mathcal {A}$ are computationally tractable (i.e. can be run on any situation *s* in polynomial time, $O(n^c)$, where *n* is some measure of the input size (|*s*|) and *c* is a constant). This constraint ensures that any intractability results we may derive for Dr. Ingenia’s AI-by-Learning problem are not an artefact of the time-complexity of running the algorithm *A* itself. Moreover, it ensures that even if human cognitive computations are all tractable (cf. ‘the tractable cognition thesis’, van Rooij, 2008; see also Frixione, 2001), our intractability results for AI-by-Learning would still hold.

We assume in our formalisation that whenever Dr. Ingenia tries to solve an instance of AI-by-Learning and searches for a program $L_A$ that encodes a human (-like or -level) *A*, there is a given upper bound *K* on the size of the program that they can in principle encode (i.e. $|L_A| \le K$). One can think of *K* as expressing the total amount of space (computer memory) that Dr. Ingenia has available to store a program. AI models these days can be very large, and we allow for their size (e.g. an ANN may have tens of millions of parameters; Krizhevsky et al., 2012). Nonetheless, they are still bounded by some size *K*. Dr. Ingenia can buy more space to work with, in which case they will have a new $K' > K$ to work with. AI engineers that claim to be able to create AGI with ML are (implicitly) assuming that their approach can work for larger and larger $K'$.

We formalise the distribution $\mathcal {D}$ as follows. A dataset *D* drawn from $\mathcal {D}$ consists of a list of situation-behaviour pairs, a.k.a. ‘samples’: $(s_1,b_1), (s_2,b_2), (s_3,b_3),..., (s_{|D|},b_{|D|})$. Without loss of generality, we model this structure with binary strings (e.g. $s = 101010101000001$ and $b = 010101111$). For any given input distribution $\mathcal {D}_n$, there is an upper bound *n* on the description length of situations. In other words, the set of situations for such an instance of the problem is defined as $S = \{0,1\}^n$. For each situation $s \in S$, there are some appropriate (i.e. human-like) behaviours $B_s \subsetneq B = \{0,1\}^m$, for some fixed *m*.

Lastly, we formalise the notion of ‘approximate’ in Dr. Ingenia’s AI-by-Learning problem. Recall that we are trying to estimate a *lower*-bound on the real-world problem of creating human-like or -level AI by ML and therefore give Dr. Ingenia ‘easier’ conditions than may apply in the real world. To this end, we will be setting an extremely low bar for what counts as ‘approximate’. On the one hand, we do not expect a guarantee that Dr. Ingenia succeeds, but merely that Dr. Ingenia succeeds with non-negligible probability (denoted $\delta (n)$, where *n* is a measure of the size/complexity of situations; see previous paragraph). Moreover, the performance of the found *A* need not have high degrees of human-likeness, but merely should perform human-like with a probability that is non-negligibly higher than chance level. Specifically, in our formalisation of AI-by-Learning, we will make the simplifying assumption that there is a finite set of possible behaviours^10^ and that for each situation *s* there is a fixed number of behaviours $B_s$ that humans may display in situation *s*. Then, $\nicefrac {|B_s|}{|B|}$ expresses chance level, and $\nicefrac {|B_s|}{|B|} + \epsilon (n)$ expresses ‘non-negligibly better than chance’.

Given the above considerations, we can now state a formalised version of AI-by-Learning:

AI-by-Learning (formal)

*Given:* An integer *K* and a way of sampling from a distribution $\mathcal {D}$.

*Task:* Find a description $L_A \in \mathcal {L}_{\mathcal {A}}$, with length $|L_A| \le K$, of an algorithm $A \in \mathcal {A}$ that with probability $\ge \delta (n)$, taken over the randomness in the sampling, satisfies:

$$\begin{aligned} \Pr _{s \sim \mathcal {D}_n} \left[ A(s) \in B_s \right] \ge \nicefrac {|B_s|}{|B|} + \epsilon (n). \end{aligned}$$

Here, $\delta (n)$ and $\epsilon (n)$ are arbitrary non-negligible functions. A function *f* is *non-negligible* if there is some *d* such that for sufficiently large *n*, $f(n) \ge {1}/{n^d}$.

### Ingenia Theorem

*[E]ven with a whole row of the largest imaginable computers to help, all the potential distributional potentialities of a whole national language cannot possibly be found in any finite time[.]*

— Margaret Masterman (1965, p. iv-19)

**Fig. 3:**
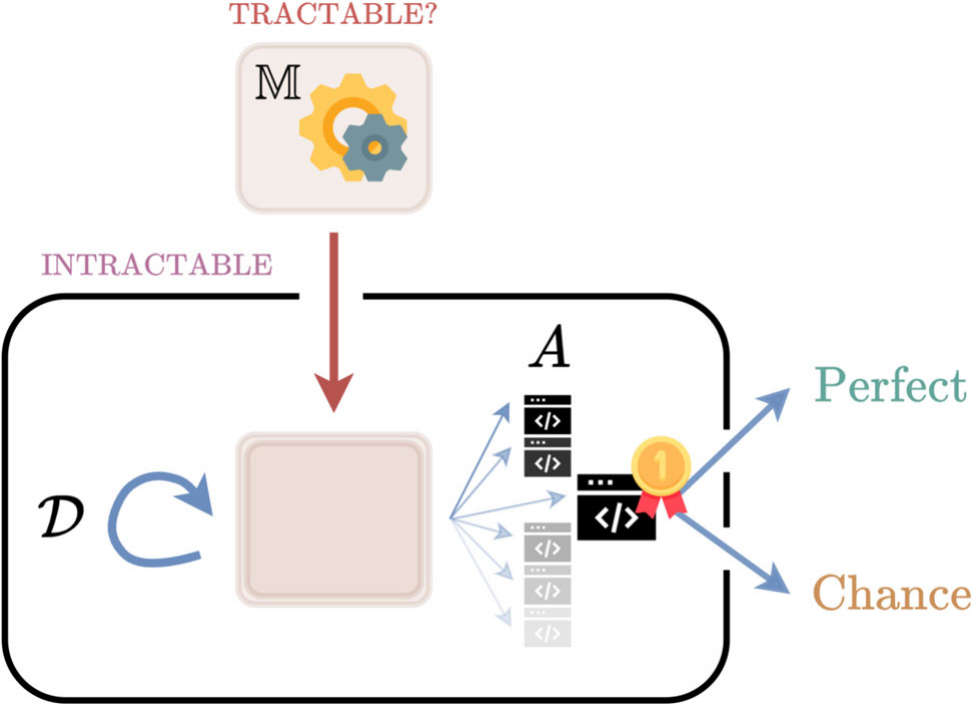
A visual illustration of the core proof idea for Theorem 2 (Ingenia Theorem): It is known that Perfect-vs-Chance is an intractable decision problem. If a tractable method $\mathbb {M}$ for solving AI-by-learning would exist, then we could use $\mathbb {M}$ to solve Perfect-vs-Chance tractably, by plugging it into a sampling-plus-decision procedure that is itself tractable, easy to construct, and together with $\mathbb {M}$ would provably solve Perfect-vs-Chance. This yields a contradiction. Therefore, we can conclude that AI-by-learning is intractable as well. See the main text for more information and the Appendix for full proof details

In this section, we present a formal proof that AI-by-Learning is intractable. For this, we use the following decision problem called Perfect-vs-Chance.^11^Perfect-vs-Chance is a somewhat unnatural problem, with no direct real-world analogue and no special connection to human cognition. It is used in our proof that AI-by-Learning is intractable (Theorem 2). The proof technique that we use comes from theoretical computer science (see Appendix for full details) and has the following logical form: If AI-by-Learning were tractable, then Perfect-vs-Chance would be tractable too. Combined with a previously known result that Perfect-vs-Chance is intractable (Theorem 1 below), we can conclude a contradiction. Figure 3 illustrates the idea.

The Perfect-vs-Chance problem is formally defined as follows:

Perfect-vs-Chance (decision problem)

*Given:* A way to sample a given distribution $\mathcal {D}$ over $\{0,1\}^n \times \{0,1\}$, an integer *k*, and the promise that one of the following two cases apply:

1. There is an efficient program *M* of size at most *k* such that $\Pr _{(x,y)\sim \mathcal {D}} [M(x) = y] = 1$
2. For any program *M* of size at most *k*, $\Pr _{(x,y)\sim \mathcal {D}} [M(x)$ $= y] \le 1/2 + 1/2^{n^{1-\delta }}$

where $0< \delta < 1$ is an arbitrary constant.

*Question:* Is (1) or (2) the case?

In the decision problem Perfect-vs-Chance, the expression $1/2^{n^{1-\delta }}$ can informally be read as ‘negligible probability’, since the denominator grows very fast. Hence, informally, the two cases listed in the Perfect-vs-Chance problem are (1) there exists a perfect and efficient program or, otherwise, (2) there exists no program that can work better than chance, even inefficiently.

Note that in the Perfect-vs-Chance problem it is *promised*, before trying to solve the problem, that for the given distribution either case (1) or (2) holds. One may intuit that, since (1) and (2) are extreme cases that are very clearly distinct, telling these two cases apart should be easy to do. However, it is not easy. In fact, it is provably *intractable* to find out in which of the two cases one finds oneself. This follows from a proof by Hirahara (2022).

#### Theorem 1

Hirahara (2022) Perfect-vs-Chance is in-tractable.^12^$$^,$$^13^

We will use Theorem 1 to prove that AI-by-Learning is intractable, too. Specifically, we will show that *if* it were possible to tractably solve AI-by-Learning, *then* it would also be possible to tractably solve Perfect-vs-Chance. Since Perfect-vs-Chance is known to be intractable (Theorem 1), by* modus tollens*,^14^ it follows that AI-by-Learning must be intractable as well. In other words, the proof will be by contradiction.

#### Theorem 2

(Ingenia Theorem) AI-by-Learning is intrac-table.

#### Proof

*(sketch)* For full details of the proof, we refer the reader to the Appendix. In this proof sketch, we present the main proof idea. See Fig. 3 for an illustration.

We prove by contradiction. Suppose that there exists a learning mechanism $\mathbb {M}$ that solves AI-by-Learning in polynomial time. We will show that then there exists a polynomial-time bounded-error probabilistic algorithm that solves Perfect-vs-Chance, contradicting intractability of Perfect-vs-Chance.

The algorithm for Perfect-vs-Chance that we construct works as follows. Take an arbitrary instance for Perfect-vs-Chance, consisting of integers *n* and *k* and a distribution $\mathcal {D}$ over $\{0,1\}^n \times \{0,1\}$. The algorithm will use a subroutine that simulates the learning mechanism $\mathbb {M}$, where $K = k$ and where the data that the mechanism $\mathbb {M}$ has sampling access to is given by the distribution $\mathcal {D}$. In this simulation, the set of situations is $S = \{0,1\}^n$ and the set of behaviours is $B = \{0,1\}$. The simulation will yield an algorithm *A* that might or might not perform well on freshly sampled situations.

After running the subroutine that simulates $\mathbb {M}$, the algorithm will evaluate the quality of the resulting learned algorithm *A* by using additional samples from $\mathcal {D}$ and counting the number of situations *s* in which the algorithm *A* returns an appropriate behaviour $b \in B_s$.^15^ As this evaluation only involves executing *A* and counting, it can be done in polynomial time.

The algorithm runs the simulation subroutine several times, and for each run of the subroutine, it evaluates the resulting learned algorithm *A*. From all of these runs, it picks the algorithm $A^{\star }$ that achieved the highest accuracy in the quality evaluation.^16^ Based on how well the most accurate learned algorithm $A^{\star }$ performs, the algorithm will give an answer for the input of Perfect-vs-Chance. If $A^{\star }$ performs non-negligibly better than chance, then the algorithm will answer Yes, and otherwise, the algorithm will answer No.

By having the algorithm run the simulation a large enough (yet polynomial) number of times, and testing the output of each simulation a large enough (yet polynomial) number of times with new samples, we can ensure that the algorithm outputs a correct answer for Perfect-vs-Chance with high probability. A careful probabilistic analysis shows that because the learning mechanism $\mathbb {M}$ performs non-negligibly better than chance on AI-by-Learning, the resulting algorithm for Perfect-vs-Chance gives the correct answer with high probability (see Appendix for full details). This contradicts the known intractability of Perfect-vs-Chance, hereby concluding the proof that AI-by-Learning is intractable.

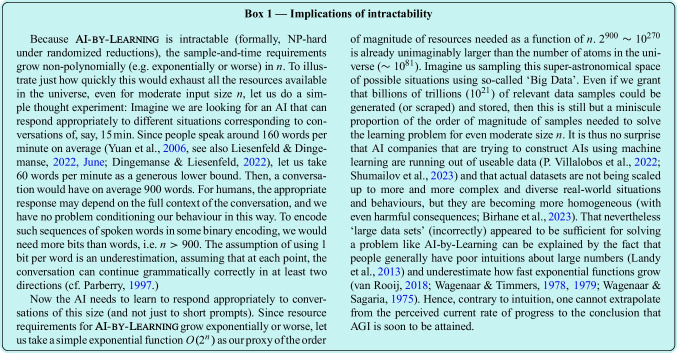



### Implications

*It is desirable to guard against the possibility of exaggerated ideas that might arise as to the powers of [AI]. In considering any new subject, there is frequently a tendency [...] to overrate what we find to be already interesting or remarkable[.]*

— Augusta Ada King, Countess of Lovelace (personal correspondence, July, 1843; Toole et al., 1998, p.186)

In the previous section, we presented a proof that Dr. Ingenia set themselves a machine learning problem for which no tractable method exists or can exist. The ‘intractability’ means that, even if the problem may be practically solvable for trivially simple situations (small *n*), any attempts to scale up to situations of real-world, human-level complexity^17^ (medium to large *n*) will necessarily consume an *astronomical* amount of resources (such as time and number of samples; see Box 1 for an illustration).

The proven intractability holds for the highly simplified and idealised model of AI-by-Learning that grants Dr. Ingenia much better conditions than apply to their real-world counterparts. As explained in Section “FormalisingAI-by-Engineering”, the intractability result holds even if Dr. Ingenia (a) can sample randomly and unbiasedly, if they so wish; (b) has data which are noiseless, without error, and uncontaminated; (c) is free to use any means or methods for producing the AI (this can include present and future machine learning approaches, but is not limited to them); (d) is only required to produce with a probability slightly higher than chance an AI that matches human behaviour slightly better than chance; (e) is guaranteed that there exists an algorithm that meets those low-bar requirements. Idealizations (a)–(e) make clear that the computational complexity of Dr. Ingenia’s problem is a gross underestimation of the true complexity of the much more messy real-world AI-by-Learning problem.

Given the proof nature of the complexity-theoretic result, the claim that we are presently on a *fast track* to inevitably produce human-like and -level AI poses a logical contradiction. Let us unpack why this is so: While there are many claims that might reasonably be based on intuitions, any claim of the inevitability of producing any desired object (or event, or state of affairs) requires a tractable procedure as a precondition. To see this, note that, without loss of generality, we can cast the problem of building human-like AI as that of searching for such an object in some space of possibilities. To claim that the search for this object is *bound to succeed in practice* is to minimally claim that one has a tractable procedure for conducting the search that provably finds the object if it exists (i.e. one has a way of performing the search in a realistic amount of time). That is, to support the *inevitability* claim one would have to put forth a set of arguments, logically and mathematically sound, to prove not only that such a tractable procedure can exist, but also that one has it. Theorem 2 (the *Ingenia Theorem*) shows that this is impossible.

Given the Ingenia Theorem, how should we interpret what is happening in practice? In practice, AIs are being continuously produced which are claimed to be either human-like and human-level AI or inevitably on a path leading there. Any AIs produced in practice, however, are produced either by tractable procedures or by cutting short a procedure that would run longer (for an unfeasible amount of time). Hence, the produced AIs necessarily fail to solve the intractable learning problem. Concretely, this means that they make lots of errors—deviating substantially from human behaviour (e.g. Bowers et al., 2022)—and fail to meet the low standard set in the Ingenia Theorem (see also the list of simplifications and idealisations (a)–(e) above). These errors cannot be contained to be small, and no matter how impressive the produced AIs may appear, they fail to capture the distribution of human behaviour even approximately.

We realise that the implications that we have drawn out from our complexity-theoretic results may appear to contradict both intuition and experiences with existing AIs. However, the pattern of observations is entirely consistent with and predictable from the Ingenia Theorem. Many AIs do seem to have truly impressive human likeness and may even sometimes fool one into thinking that they have agency or are sentient.^18^ Moreover, the field of AI-as-engineering has a habit of interpreting (or selling) ‘better than the state of the art’ as ‘good accuracy’, but our results imply that no matter how much ‘better’ AI gets, it will be off by light-years, wrong in exponentially many situations. This kind of self-fooling is possible in part because:

The prioritization of performance values is so entrenched in the field that generic success terms, such as ‘success’, ‘progress’, or ‘improvement’ are used as synonyms for performance and accuracy [...] However, models are not simply ‘well-performing’ or ‘accurate’ in the abstract but always in relation to and as quantified by some metric on some dataset” (Birhane et al., 2022).

However, the Ingenia Theorem implies that if one were to test these AIs rigorously and unbiasedly for human-likeness, it would quickly become evident that they behave qualitatively differently from humans. That is, if you think your AI is very human-like, then you are not testing it critically enough (cf. Bowers et al., 2023).

Unsurprisingly given our theoretical results, we see exactly this play out in practice: AIs appear human-like in non-rigorous tests, but the likeness is debunked when more rigorous tests are made (e.g. Adolfi, et al., 2023; Dentella et al., 2023). For instance, claims of abilities emerging with the scaling up of models are often revealed to be trivial products of the researcher’s choice of metric (Schaeffer et al., 2023). This back and forth between claims of human-likeness and debunking (cf. Mitchell, 2021) will keep happening if the field does not realise that AI-by-learning is intractable, and hence any model produced in the short run is but a ‘decoy’.

This is especially troubling since more and more people are taking AI systems to be candidate models of human cognition (Frank, 2023a, b; Hardy et al., 2023; Mahowald et al., 2023; Tuckute et al., 2023) or even as replacements for humans. For instance, AI systems are seen as a replacement for participants in psychological experiments (Dillion et al., 2023); but see also: (Crockett & Messeri, 2023; Harding et al., 2023) or as a replacement for workers (Eloundou et al., 2023; Rose, 2023; Semuels, 2020). By now, though, it is clear that this is only possible at the cost of an exponential increase of hidden, poorly paid and poorly treated workers (McCarty Carino & Shin, 2023; Roberts et al., 2023). Such replacements are a clear case of ‘map territory confusion’ and with a poor map at that. This may seem to make sense if one believes that the AIs approximate human behaviour (though even then it is not a sufficient condition, Guest & Martin, 2023), but as we explained above, the AIs do not actually approximate human behaviour. By nevertheless taking the AIs as cognitive models, we—as a field—distort our view of cognition, and it makes our cognitive science theoretically weaker.

This argument applies not only to AIs mistaken for models of (all of human) cognition, but for models of substantive cognitive capacities, like language, problem-solving, reasoning, analogizing, or perception (Cummins, 2000; van Rooij & Baggio, 2021). This is because these substantive cognitive capacities are already human-level in the sense of being domain general and able to operate on a set of situations which is in principle unbounded. Therefore, these substantive cognitive capacities also cannot be modelled through trivial computations or with a fixed problem space and instead require computational models that meet the minimal computational assumptions as specified in Section “FormalisingAI-by-Engineering”, and in fact even the stronger assumptions discussed there. Additionally, no piecemeal or divide-and-conquer approach to making models of human-level cognition (or these substantive capacities) can be tractable, as that would lead to a contradiction: Assume it were possible to tractably make approximate models of subcapacities, such that one could make piecemeal models of human cognition. Then, one would not be able to put them back together tractably in order to account for all of human cognition (or substantive capacities), because if one were able to, then one would have a tractable procedure for modelling all of cognition, which is an intractable problem (see also Rich et al., 2021).

## ACT 2: Reclaiming the AI Vertex

*Computers as such are in principle less crucial for cognitive science than computational concepts are.*

— Margaret A. Boden (2008, p. 14)

Based on our analysis, we reject the view and associated project that we term ‘makeism’. Makeism has not been characterized before, but we argue that its adoption is the reason why, historically, the use of AI to understand cognition fizzled out and why it will do so again if we do not change our present course. See Box 2 for a definition of makeism; in other words, all makeists think that building cognition is sufficient for being able to explain it (*b*), and some think that this building is also necessary (*c*). The necessity claim especially reveals the implicit assumption that it is possible to build cognition (*a*). While some things can be understood by making them, it will not work for human-like or -level cognition, for one because this cannot plausibly be (re)made through engineering (i.e. (*a*) is false; see the Ingenia Theorem).

At this point, the reader may wonder: if we indeed cannot (re)make cognition—or coherent substantive parts of cognition—computationally, then is AI theoretically useless for cognitive science? No: computationalism can be theoretically productive even if makeing is futile. In this section, we explain how the notion of computation can help to challenge and constrain, and thereby inform, theories of cognition in a way that steers clear of makeism. This will also allow us to reclaim ‘AI’ as one of the cognitive sciences, i.e. one of the vertices in the hexagon (Fig. 1). In our opinion, this is vital for retaining (or restoring) cognitive science’s theoretical health and preventing (further) distortions of our understanding of human cognition.

### What Not to Reclaim

As we explained in the Introduction, AI was initially conceived as a theoretical tool for cognitive science, and an active segment of work in cognitive science was understood as being part of AI. AI as a field originally included the use of computational models to study the human mind (variously referred to as cognitive simulation (Lehnert, 1977), information processing psychology (Newell, 1970; Simon, 1983), computational psychology (Boden, 1988, 2008), or theoretical psychology (Hünefeldt & Brunetti, 2004; Longuet-Higgins, 1982; Newell, 1970).

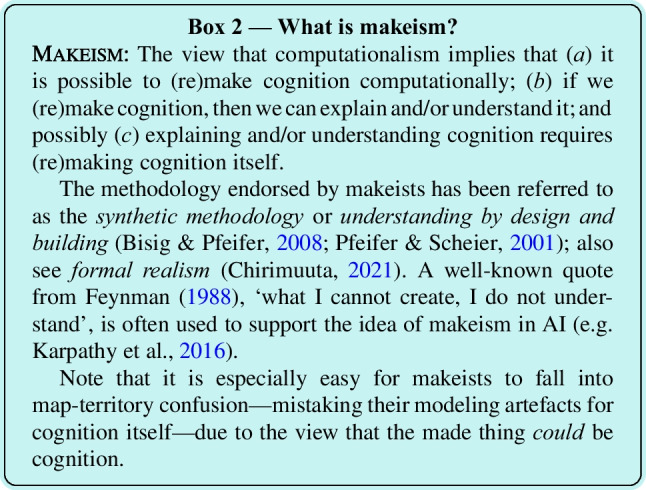



At present, this perspective on AI is largely forgotten. By looking at the history of those conceptions of AI and why they fell out of favour, we can see both the attraction of AI as cognitive science and the problems with the original vision, which we do not want to reclaim.

First, let us consider makeism part (*a*) (see Box 2). As noted, the makeist project only makes sense if part (*a*) is really true, i.e. if cognition *can* be programmed into a computer. This seems to have been taken for granted by many, for example by Simon, who wrote:

It is not my aim to surprise or shock you. [...] But the simplest way I can summarize is to say that there are now in the world machines that think, that learn and create. Moreover, their ability to do these things is going to increase rapidly until–in a visible future–the range of problems they can handle will be coextensive with the range to which the human mind has been applied.

Herbert Simon on The General Problem Solver in 1957, as quoted in Norvig (1992, p. 109)

Here we see the forerunner of the current idea that computationalism implies the practical realizability of thinking machines/simulations on par with human-level or -like cognition. This idea is expressed clearly in Feigenbaum and Feldman (1963) early characterization of AI (as quoted in Meinhart, 1966, emphasis added):

Researchers in the (artificial intelligence) field hold to the working hypothesis that human thinking is wholly information-processing activity within the human nervous system; that ultimately, these information processes are perfectly explicable; that the road to this explication lies in observation, experimentation, analysis, modelling, model validation, et cetera; *and that digital computers, being general information processing devices, can be programmed to carry out any and all of the information processes thus explicated*.

The last item on the list makes explicit the assumption that computationalism *implies* the practical realizability of thinking machines/simulations on par with human-level or -like cognition. And since it suddenly seemed possible to re-create aspects of cognition using computers, many early cognitive scientists enthusiastically began trying to do so. For example, consider representative comments from the late 70s, from a book by Lehnert (1977).

If experiments cannot be designed to isolate the variable factors of a proposed theory, the [experimental] psychologist can go no further. Problems concerning human cognitive processes are difficult to study within the paradigm of experimental psychology for precisely this reason. [...] What experiment can be designed to help us understand how people are able to answer simple questions like “What’s your name?” [...] Natural language processing can be productively studied within the artificial intelligence paradigm. If we construct a process model designed to account for a particular language task [...], then we can write a computer program to implement that model. By running that program, we can see where the model is weak, where it breaks down, and where it appears competent. [...] The interesting failures are those that occur because the process model underlying the program failed to recognize some critical problem or failed to handle some problem adequately (Lehnert, 1977, pp. 40-41).

If our reading of Lehnert is correct, this expresses, or at least encourages, makeism. Specifically, it reflects parts (*a*) and (*b*), with the idea that interesting parts of cognition can be simulated in computers and that we will gain understanding in this way. Furthermore, Lehnert leaves the door open to makeism part (*c*); it is unclear whether she herself thinks that simulating natural language is necessary for explaining it, but one could draw that conclusion.

The problem, of course, is that (*a*) is false, and without it, makeism (*b*) and (*c*) no longer reflect a promising strategy for cognitive science, but rather a research program doomed to fail. Indeed, in light of our demonstration that the task of creating cognition in computers is unfeasible, it is not surprising that among early researchers pursuing this project, enthusiasm waned and many people moved on.^19^

As AI technology has exploded, makeism is enjoying a renaissance. However, for cognitive science, the engineering approach worsens theoretical understanding because any artefacts we could make in the short run would be gross distortions or ‘decoys’ at best. As Neisser wrote, ‘[t]he view that machines will think as man [sic] does reveals misunderstanding of the nature of human thought’ (Neisser, 1963, p. 193). Sixty years later, the risk of being misled by decoys is even greater, but we can better demonstrate the problem through the complexity theoretical arguments laid out in the previous Section (“ACT 1: Releasing the Grip”).

To abandon all of the tools and concepts that AI provided, however, is to throw the baby out with the bath water.^20^ Hence, we want to reclaim much of the early conception of AI as a part of cognitive science, but without encouraging makeism. If we reconsider Lehnert’s argument for using AI, we see many correct, important insights:

1. That experimental psychology is limited in its ability to study cognition (hence the creation of cognitive science as an interdisciplinary field).
2. That cognition can be productively studied within the AI paradigm.
3. That models’ problems are theoretically informative.

These insights are all worth reclaiming; the community just made one seemingly small inferential miss-step that has caused a lot of problems. Makeism is not a forced move. Hunt pointed this out early on, arguing that

Computer programming seems to be a more appropriate tool for studying the broad implications of a proposal for how one should think than for realizing a testable model of how one does think (Hunt, 1968, p. 160).

Computationalism without makeism is still theoretically fruitful. We explain how next.

### Theory Without Makeing

*[P]rinciples from computer science and engineering can be, if done carefully, imported into how we carve [...] nature at its joints.*

— Olivia Guest and Andrea E. Martin, (2023, p. 221)

How may computationalism help cognitive science advance if not through makeism? Core to the non-makeist enterprise is the realisation that computationalism primarily aids cognitive science by providing conceptual and formal tools for theory development and for carefully assessing whether something is computationally possible or not, in principle and in practice. This paper is itself an example; nowhere in this paper did we (try to) make a computational replica of cognitive capacities. Yet, we were able to use a computationalist framework to make substantial steps in reclaiming AI for cognitive science. The remainder of this section will give further examples of how AI as theoretical psychology or computational cognitive science can be pursued productively and soundly.^21^

As a disclaimer, we note that research in cognitive science often cannot be cleanly divided into makeist and non-makeist. We have not seen makeism clearly distinguished from computationalism in the literature before (see Box 2), and so cognitive scientists will generally not have thought about it explicitly, let alone clarified the nature of their work. Hence, when we cite papers as examples, we wish to highlight the non-makeist readings of some of the arguments made, but without implying that there are no problematic traces of makeism in the original texts.

#### Levels of Explanation

Formalisms and concepts from computer science allow us to conceptually distinguish between cognitive processes (algorithms), the capacities they realise (the problems that they solve), and their physical implementations (chemical, biological, interactive, etc.). This conceptual distinction, also often referred to as Marr’s levels (Marr, 1982), is theoretically productive, especially when pursued in a non-makeist fashion.

The distinction between levels is conceptually useful in general, but also brings specific benefits when we want to formalise and reason about our theories, as we explain next.

#### Capacities as Problems

Within the levels-framework, the approach known as compu-tational-level modelling has a strong tradition in cognitive science (with debates on its proper interpretation continuing to this day; Blokpoel, 2018; Cooper & Peebles, 2018; Peebles & Cooper, 2015). This approach allows us to conceptually engineer cognitive capacities as ‘computational problems’ and to model them formally (see e.g. Blokpoel & van Rooij, 2021; van Rooij & Baggio, 2021; van Rooij & Blokpoel, 2020) without needing to commit to specific assumptions at the algorithmic or implementation levels (other than computability and tractability; more in the next subsection). This is especially useful since—as argued throughout this paper—we do *not* know how to computationally realise substantive cognitive capacities such as human-level perception, reasoning, memory, categorisation, decision-making, problem-solving, language, analogising, communication, learning, and planning. Yet, as cognitive scientists, we *do* want to make progress in developing a theoretical understanding of these capacities.

Computational modelling of capacities can help us to make our assumptions precise and explicit, and to draw out their consequences, without the need to simulate the postulated computations (though simulations have their uses; more on that next). For instance, with formal computational-level models and mathematical proof techniques at hand, one can critically assess claims of explanatory adequacy (Blokpoel & van Rooij, 2021; Egan, 2017; van Rooij & Baggio, 2021), claims of intractability (Adolfi, et al., 2023), claims of tractability (Kwisthout & van Rooij, 2020; van Rooij et al., 2008), claims of competing theories (Blokpoel & van Rooij, 2021), claims of evolvability (Rich et al., 2020; Woensdregt et al., 2021), and claims of approximability (Kwisthout & van Rooij, 2013; Kwisthout et al., 2011).

#### Algorithms and Simulations

Similarly, algorithmic- and implementation-level models can be postulated and critically assessed using computational tools. While this can sometimes be done analytically, more often computer simulations prove useful for these types of (complex and dynamic) models. Using computer simulations, for example, one can assess claims about possible functioning under network damage (Guest et al., 2020), claims of explanatory scope and adequacy (van de Braak et al., 2021; Adolfi et al., 2023), claims of approximation (Blokpoel & van Rooij, 2021, Chapter 8), claims of ruling out possible so-called neural codes (Guest & Love, 2017), and claims of mechanistic possibilities (Bartlett et al., 2023; ten Oever & Martin, 2021).

Importantly, this use of simulations is to be distinguished from makeist uses of simulation that confuse the models (explanans) for the thing modelled (explanandum) and/or take the simulation results to directly imply something about ‘how things work’ in the real world (e.g. for real-world brains, cognition, or behaviour). Instead, non-makeist computer simulations are theoretical tools that can demonstrate proof of concept or demonstrate the in-principle (im)possibility of phenomena arising from the theorised constructs and hypothesised mechanisms. Computer simulations support and extend a scientist’s thinking capacity and enable computerised ‘thought experiments’ (Cooper, 2005) to reason through ‘what ifs’ and answer questions like ‘how possibly’.^22^ These simulations—as indeed any models that the cognitive scientist could use—are necessarily abstract and idealised; this is unproblematic, though, as long as the scientist recognises it and takes care to draw only those inferences which are really warranted by the model.

#### Underdetermination

A general theoretical property that follows from computationalism is that cognitive capacities are multiply realisable, in several ways (Chirimuuta, 2021; Guest & Martin, 2023; Hardcastle, 1995, 1996). van Rooij and Baggio (2021) use a sorting problem as a simple illustration. Sorting can be done by bubble sort, insert sort, or any of a whole host of distinct sorting algorithms (Knuth, 1968) which in turn can be physically realised by brains, computers, water pipes, or even distributed over people (see, e.g. Fig.  1 in van Rooij & Blokpoel, 2020; and Box 1 in van Rooij & Baggio, 2021). This shows how, first, one and the same problem can be computed by different algorithms, and second, one and the same algorithm can be physically realised in different ways. This implies that we are dealing with massive underdetermination of theory by data: i.e., if we observe behaviours consistent with a computational-level theory, we cannot infer which algorithms or neural processes underlie the behaviour.

The computational lens helps us to appreciate the degree of underdetermination we face. In standard experimental cognitive psychology, often two or a handful of different theories are compared and tested empirically ‘against each other’. But the principle of computational multiple realisability shows that for any given behaviour, there may be $|\mathcal {A}| \times |\mathcal {I}|$ many possible algorithmic-implementational theories. This means that some inferential practices in computational cognitive (neuro)science are highly problematic (Guest & Martin, 2023). Moreover, computational-level theories are also themselves underdetermined by data, because any finite set of observations is also consistent with infinitely many functions (capacities).^23^ This means that all computational-level theories are—and remain—conjectural. Nonetheless, we can do some things to evaluate and adjudicate between them.

#### Computational Realisability

Underdetermined computational-level theories can be constrained by the computationalist requirement that the problems and processes they postulate must be computationally realisable—by the cognitive system under study, *not* by scientists—both in principle and in practice. In-principle realisability is also known as computability; a problem is *computable* if there can exist at least one algorithm for computing it. In-practice realisability is also known as tractability; a problem is *tractable* if there can exist at least one tractable^24^ algorithm for computing it.

Given that computational-level theories often formalise capacities as problems (or equivalently functions) while remaining agnostic about how these problems are computed, they can on occasion postulate problems that are uncomputable or intractable; in fact, this happens regularly. This provides the opportunity to critically reflect on the theory and, if possible, to find a minimal revision that renders the theory minimally computable and tractable while preserving the core intuitions and motivations behind the theory. This process can yield new knowledge, ideas, and research trajectories for cognitive scientists (cf. Adolfi et al., 2023). For example, it may yield new theoretical interpretations or predictions that can be used to further assess the explanatory and empirical adequacy of the (revised) theories. Alternatively, if a theory cannot be successfully revised, this can be a sign that it is time to question its initial motivation and to go back to the drawing board. The process thereby allows us to sculpt otherwise underdetermined theories so as to learn more about how cognition could or could not work (Blokpoel, 2018). This is no magic bullet, however; underdetermination of theory by data cannot be eliminated, and any ways of dealing with it will remain necessarily incomplete (cf. Adolfi et al., 2023; Devezer, 2024; Rich et al., 2021).

#### Slow (Computational Cognitive) Science

All of this may seem excruciatingly slow compared to the apparent speed of progress in today’s machine learning approaches to AI. But to genuinely make progress, we need to go this slowly, and in fact, we cannot go any faster (Adolfi & van Rooij, 2023; Rich et al., 2021). There is just no way to proceduralise or automate either the creation of minds or the explanation of minds. The way to make progress is through the meticulous development of theoretical ideas, informed by formal and computational modelling, drawing out limitations, consequences, and building solid knowledge along the way. In other words, what we advocate is more theoretical thinking (see also Guest, 2024; Guest & Martin, 2021; van Rooij & Baggio 2020) and less (unthinking) machine learning or less confusion between machine learning and theory (cf. Andrews, 2023). Then, AI can be a useful theoretical tool for cognitive science and regain its rightful place in the interdisciplinary hexagon.

## Conclusion

The thesis of computationalism implies that it is possible in principle to understand human cognition as a form of computation. However, this does not imply that it is possible in practice to computationally (re)make cognition. In this paper, we have shown that (re)making human-like or human-level minds is computationally intractable (even under highly idealised conditions). Despite the current hype surrounding ‘impending’ AGI, this practical infeasibility actually fits very well with what we observe (for example, running out of quality training data and the non-human-like performance of AI systems when tested rigorously).

Many societal problems surrounding AI have received thorough treatment elsewhere. Our focus here has been on a different—but not unrelated—problem, namely that AI-as-engineering has been trespassing into cognitive science, with some people drawing overly hasty inferences from engineered AI systems to human cognition. This is a problem because any such system created now or in the near future is a mere decoy when our goal is to understand human cognition, and treating it as a substitute for human cognition for scientific purposes will only confuse and mislead us.

Early cognitive scientists rightly recognised the tremendous potential of AI as a theoretical tool, but due to widespread, implicit makeist elements, AI and cognitive science became increasingly dissociated over time. Now, interest in AI among cognitive scientists is enjoying a renaissance—but the interest seems to be in the wrong type of AI, namely AI-as-engineering, which distorts our understanding of cognition and cognitive science. Accordingly, the time is apt to reclaim AI-as-theoretical-psychology as a rightful part of cognitive science. As we have argued, this involves embracing all the valuable tools that computationalism provides, but without (explicitly or implicitly) falling into the trap of thinking that we can or should try to engineer human(-like or -level) cognition in practice.

## Appendix

This appendix contains some additional technical material, leading up to (and including) the detailed proof of Theorem 2. Our aim in this appendix is to make the proof accessible to an audience that has a general (theoretical) computer science background.

We will begin with some brief reminders of various notions from computational complexity theory needed to follow the proof. We will assume that the reader is familiar with basic notions such as the complexity classes $\text {P}$ and $\text {NP}$, polynomial-time (many-to-one) reductions, and the notions of $\text {NP}$-hardness and -completeness. For more details on these notions, we refer to textbooks on the topic (e.g. Arora & Barak, 2009).

***Probabilistic Computation*** The first notion that we will recap is that of probabilistic algorithms and the complexity class $\text {BPP}$. Intuitively, a probabilistic algorithm has access to a random bit generator and can use these random bits in its computation. This can be formalized using the notion of probabilistic Turing machines. The result of this is that the running time and the output of the algorithm are both random variables.

The complexity class $\text {BPP}$ contains all decision problems that can be solved by a bounded-error polynomial-time probabilistic algorithm. This means the following. The running time of the algorithm should be upper bounded by a polynomial of the input size (i.e. regardless of the randomness, the algorithm should halt within polynomial time). Moreover, the output of the algorithm should be correct with probability at least $\nicefrac {2}{3}$. In other words, the algorithm may make mistakes (in the sense of outputting the wrong answer), as long as these occur with low probability. (For more details on probabilistic algorithms and the class $\text {BPP}$, see, e.g. Arora & Barak, 2009, Chapter 7).

A (bounded-error) randomized polynomial-time reduction from one decision problem $P_1$ to another decision problem $P_2$ is a probabilistic polynomial-time algorithm that takes an input *x* for $P_1$ and produces an input *y* for $P_2$. The algorithm should satisfy the property that (1) if $x \in P_1$, then the probability that $y \in P_2$ is at least $\nicefrac {2}{3}$, and (2) if $x \not \in P_1$, then the probability that $y \not \in P_2$ is at least $\nicefrac {2}{3}$. (Remember that *y* is a random variable.) In other words, the reduction should be correct (in the sense that the produced instance is a yes-instance if and only if the original instance is a yes-instance) with probability at least $\nicefrac {2}{3}$.

***Promise Problems*** A further notion that plays a role in the proof of Theorem 2 is that of promise problems. Intuitively, a promise problem involves the promise that the input satisfies a certain property. An algorithm solving a promise problem is only evaluated on inputs that satisfy this promised property. This can be formalized as follows. The inputs satisfying the promised property are captured by a set $P \subseteq \Sigma ^{*}$ of strings, over a given alphabet $\Sigma$. Then, the yes- and no-inputs of the problem are specified by two sets $P_{\text {yes}}, P_{\text {no}} \subseteq P$ such that $P_{\text {yes}} \cap P_{\text {no}} = \emptyset$ and $P_{\text {yes}} \cup P_{\text {no}} = P$. That is, the sets $P_{\text {yes}}, P_{\text {no}}$ partition *P*. An algorithm solving the problem then gets an input $x \in P$ and has to decide if $x \in P_{\text {yes}}$ or $x \in P_{\text {no}}$. (When dealing with promise problems, the notions of reductions have to be modified accordingly, in a straightforward manner.)

***Sampling*** Another notion playing a role in the proof of Theorem 2 is that of problems where one is given access to a sampling mechanism from some distribution $\mathcal {D}$. (This notion originates and features prominently in the theory of probably approximately correct (PAC) learning.) AI-by-Learning is a problem where this is the case. For such problems, the assumption is that there is some fixed probability distribution $\mathcal {D}$ (over data points whose description is of a given size *n*), but we do not know what distribution it is. Nevertheless, algorithms are given a way of sampling from this distribution $\mathcal {D}$, in the form of a (probabilistic) oracle. The requirement on the algorithm is that it produces an output that satisfies a given property with high probability, where the randomness ranges over the randomness in the sampling. Typically, this property depends on the distribution $\mathcal {D}$. Such problems thus involve a worst-case interpretation over possible distributions—that is, the algorithm should satisfy the requirements regardless of what the actual distribution $\mathcal {D}$ is (for more details on PAC learning, see, e.g. Kearns & Vazirani, 1994).

***Probability Theory*** The proof of Theorem 2 involves some probability-theoretic analysis. We will assume the reader to be familiar with basic notions from probability theory. (For a compact overview of basic probability-theoretic notions, see, e.g. 2009, Appendix A.2.) The following well-known statements will be used in the proof, which we will briefly overview here.

### Proposition

(Union bound) Let $A_1,A_2,\dotsc$ be a countable number of events. Then, $\Pr [\bigcup \nolimits _{i} A_i] \le \sum \nolimits _{i} \Pr [A_i]$.

### Proposition

(Markov’s inequality) Let *X* be a nonnegative random variable and let $a > 0$. Then, $\Pr [X \ge a] \le \nicefrac {\boldsymbol{E}[X]}{a}$, where $\boldsymbol{E}[X]$ is the expected value of *X*.

### Proposition

(Hoeffding’s inequality) Let $X_1,\dotsc ,X_n$ be independent random variables such that $0 \le X_i \le 1$ for all *i*. Let $S_n$ be the sum of these random variables, and let ${E}[S_n]$ be the expected value of this sum. Then, for any $t > 0$, $\Pr [S_n - \boldsymbol{E}[S_n] \ge t] \le \boldsymbol{e}^{-2t^2/n}$.

***The Proof*** With the above notions in place, we will then turn to the detailed proof of Theorem 2, which captures the intractability of AI-by-Learning under the widely held assumption that $\text {NP} \not \subseteq \text {BPP}$ (see, e.g. Arora & Barak, 2009, Chapter 7).

### Theorem 2

(Ingenia Theorem) If there is a learning mechanism that solves AI-by-Learning in polynomial time, then $\text {NP} \subseteq \text {BPP}$.

### Proof

Suppose that there exists a learning mechanism $\mathbb {M}$ that solves AI-by-Learning in polynomial time. This means that there exist non-negligible functions $\delta ,\epsilon$ such that when dealing with situations of description length *n*, regardless of the distribution $\mathcal {D}_n$ that the mechanism $\mathbb {M}$ can sample from, it learns an algorithm *A* that with probability at least $\delta (n)$ satisfies that $\Pr _{s \sim \mathcal {D}_n} [ A(s) \in B_s ] \ge |B_s| / |B| + \epsilon (n)$.^25^ Let *d* be such that $\delta (n) \ge 1 / n^d$ for sufficiently large *n*, and let *e* be such that $\epsilon (n) \ge 1 / n^e$ for sufficiently large *n*.

We will show that then $\text {NP} \subseteq \text {BPP}$, by showing that there exists a polynomial-time probabilistic algorithm that solves Perfect-vs-Chance with (two-sided) bounded error. Since Perfect-vs-Chance is $\text {NP}$-hard (under randomized reductions), this suffices to show that $\text {NP} \subseteq \text {BPP}$, by the following argument. Since Perfect-vs-Chance is $\text {NP}$-hard, there is a polynomial-time randomized reduction from any problem *Q* in $\text {NP}$ to Perfect-vs-Chance. Having a bounded-error polynomial-time probabilistic algorithm for Perfect-vs-Chance then allows us to construct a similar such algorithm for *Q*, by composing the reduction and the algorithm for Perfect-vs-Chance. Therefore, we can solve any problem in $\text {NP}$ in probabilistic polynomial-time with bounded two-sided error, showing that $\text {NP} \subseteq \text {BPP}$.

The algorithm for Perfect-vs-Chance works as follows: Take an arbitrary instance for Perfect-vs-Chance, consisting of integers *n* and *k* and a distribution $\mathcal {D}$ over $\{0,1\}^n \times \{0,1\}$ (in the form of a circuit *C* that takes as input a string specifying random bits and outputs elements of $\{0,1\}^n \times \{0,1\}$).

The algorithm will use a subroutine that simulates the learning mechanism $\mathbb {M}$, run on a setting where situations are (described using strings) of length *n*, where $K = k$ and where the data that the mechanism $\mathbb {M}$ has sampling access to is given by the distribution $\mathcal {D}$. In particular, this works as follows: The set *S* of situations is the set $\{0,1\}^n$, and the set *B* of behaviors is $\{0,1\}$. Every time that the mechanism $\mathbb {M}$ asks for a data sample (*s*, *b*) consisting of a situation $s \in S$ with a corresponding appropriate behavior $b \in B_s$, the simulation of $\mathbb {M}$ produces a random string *r* that is fed as input to the circuit *C*, resulting in an element $(s,b) \in \{0,1\}^n \times \{0,1\}$, which is given to the mechanism $\mathbb {M}$ as data sample. The simulation of the learning mechanism $\mathbb {M}$ will yield a learned algorithm *A*, which is returned as output of the subroutine. Moreover, since the learning mechanism runs in polynomial time, such a simulation can also be done in polynomial time.

After running the subroutine that simulates $\mathbb {M}$, we will evaluate the quality of the resulting learned algorithm *A* by using additional samples from $\mathcal {D}$ and counting on how many of these situations *s*, the algorithm *A* returns an appropriate behavior $b \in B_s$. In particular, we will use $L_1$ additional samples, where the exact value of $L_1$ is to be specified later.

The algorithm runs the simulation subroutine $L_2$ times, where the exact value of $L_2$ is to be specified later. For each run of the subroutine, it evaluates the resulting learned algorithm *A* as described above. From all of these runs, it picks the algorithm $A^{\star }$ that achieved the highest accuracy in the quality evaluation. Let $\rho$ be the fraction of data points in the evaluation phase (i.e. situations *s*) for which $A^{\star }$ provided an appropriate behavior $b \in B_s$.

Based on the observed correctness rate $\rho$ of the learned algorithm $A^{\star }$, the algorithm will give an answer for the input of Perfect-vs-Chance. If $\rho \ge 1/2 + 1/n^d$, then the algorithm will answer Yes, and otherwise, the algorithm will answer No.

We will ensure that $L_1$ and $L_2$ are polynomial in *n*, so this algorithm runs in polynomial time. Let us analyze the error probability of the algorithm for Perfect-vs-Chance. We will show that it outputs the correct answer with probability at least 2/3.

Suppose that the instance of Perfect-vs-Chance is a yes-instance. In this case, because by the promise of Perfect-vs-Chance there exists an efficient algorithm that makes no errors at all, we know that there exists an algorithm *A* with description length $|L_A| \le K$ such that $\Pr [ A(s) \in B_s ] \ge \nicefrac {|B_s|}{|B|} + \epsilon (n)$— call this property ($\star$). Therefore, we have the guarantee that in each single simulation of $\mathbb {M}$, with probability at least $\delta (n)$, the learned algorithm *A* satisfies ($\star$).

We will consider the probability that the algorithm *A* learned in any single simulation achieves a quality evaluation that is $< 1/2 + 1/n^e$. Suppose that *A* satisfies ($\star$). Then, the probability that on $L_2$ independently drawn samples the algorithm *A* outputs the incorrect answer at least $(1/2 - 1/n^e) \cdot L_2 + 1$ times, by Markov’s inequality, is at most $1 - (L_2/2 - L_2/n^e + 1)^{-1}$. This means that the probability that the observed correctness rate of the learned algorithm *A* (based on $L_2$ new samples) is at least $1/2 + 1/n^e$ is at least $(L_2/2 - L_2/n^e + 1)^{-1}$. By ensuring that $L_2 \ge n^e$ (which we will do), we get that this probability is at least $2/L_2$. Since *A* satisfies ($\star$) with probability at least $\delta (n)$, we get that the observed correctness rate of *A* is at least $1/2 + 1/n^e$ is at least $2\delta (n)/L_2 \ge 1/(L_2 n^d)$, for sufficiently large values of *n*.

Next, we consider the probability that all algorithms *A* learned in the $L_1$ simulations of $\mathbb {M}$ achieve a quality evaluation that is $< 1/2 + 1/n^e$. By Hoeffding’s inequality, this probability is less than $\boldsymbol{e}^{-2 L_1/(L_2 n^{d})^2}$. By ensuring that $L_1 \ge (L_2 n^{d})^2$ (which we will do), we get that this probability is less than $\boldsymbol{e}^{-2} \le 1/3$. This concludes the argument that if the instance of Perfect-vs-Chance is a yes-instance, then the algorithm will output Yes with probability at least 2/3.

Conversely, suppose that the instance of Perfect-vs-Chance is a no-instance. In this case, we know that regardless of which learned algorithm *A* any simulation of $\mathbb {M}$ outputs, it holds that $\Pr _{s \sim \mathcal {D}_n} [ A(s) \in B_s ] \le 1/2 + 2^{-\sqrt{n}}$.

We will consider the probability that the algorithm *A* learned in any single simulation achieves a quality evaluation that is $< 1/2 + 1/n^e$. Then the probability that on $L_2$ independently drawn samples the algorithm *A* outputs the correct answer at least $(1/2 + 1/n^e) \cdot L_2$ times, by Hoeffding’s inequality, is at most $\boldsymbol{e}^{-2t^2/L_2}$ for $t = L_2/2 + L_2/(n^e) - L_2/2^{\sqrt{n}}$. Since $2^{\sqrt{n}} \ge n^e$ for sufficiently large values of *n*, we get that this probability is at most $\boldsymbol{e}^{-L_2/2}$.

Next, we consider the probability that all algorithms *A* learned in the $L_1$ simulations of $\mathbb {M}$ achieve a quality evaluation that is $< 1/2 + 1/n^e$. By the union bound, this probability is at most $L_1 \boldsymbol{e}^{-L_2/2}$. By ensuring that $L_1 \le \boldsymbol{e}^{L_2/2}/3$ for sufficiently large values of *n* (which we will do), we get that if the instance of Perfect-vs-Chance is a no-instance, then the algorithm will output No with probability at least 2/3.

What remains is to fix values of $L_1$ and $L_2$ that are polynomial in *n*, and that (for sufficiently large values of *n*) satisfy the conditions that $L_2 \ge n^e$, $L_1 \ge (L_2 n^{d})^2$, and $L_1 \le \boldsymbol{e}^{L_2/2}/3$. We let $L_1 = n^{2de}$ and $L_2 = n^e$, which satisfies all constraints. $\square$

## Footnotes

1 From this perspective, domain-general AI and human-level AI collapse, motivating our and others’ usage of the term AGI to refer to either; see Table 1. As we note later, our arguments will also have implications for human-level AI which is not necessarily human-like.

2 Fiorenza (1993) coined ‘kyriarchy’ as an intersectional (Crenshaw, 1989; Osborne, 2015) generalisation of the notion of ‘patriarchy’, i.e. a complex system of oppressions that include (intersections of) racism, sexism, homophobia, transphobia, ableism, etc. For an application of the concept in technology, see ‘anti-oppressive design’ by Smyth and Dimond (2014), and for an application in ‘social justice in climate change adaptation’, see Osborne (2015).

3 Here, cognitive science is defined as the inter- or multidisciplinary study of cognition.

4 See also Lighthill’s (1973) report on the state of AI research and McCorduck’s (McCorduck, 2019) history.

5 See also ‘computational psychology’ as used by Boden (1988) and ‘theoretical psychology’ as used by Longuet-Higgins (Hünefeldt & Brunetti, 2004).

6 Importantly, computationalism is to be conceptually distinguished from computerism, cognitivism, or any commitments to specific computational architectures (see, e.g. Chalmers, 2011; Dietrich, 1994).

7 This analytical strategy takes inspiration from Rich et al. (2021).

8 Here and throughout, we use ‘AGI’ in the sense of (domain-general) human-like or -level AI (cf. Fig. 1).

9 We will develop a formal definition of AI-by-Learning: below, including a weak formal constraint corresponding to approximately human-like behavior.

10 Given the assumption of productivity of mind (Fodor & Pylyshyn, 1988; Fodor, 2005), this is a gross underestimation of human potential.

11 This problem was introduced and proven intractable by Hirahara (2022), but they did not give it a name. We have chosen a name that suffices for our purposes and so as to optimise intuitiveness.

12 Formally, NP-hard under randomised polynomial-time one-query reductions. See Hirahara (2022) for more details. The reduction proves that the problem is not tractable (i.e. computable in polynomial time) unless $\text {NP} \subseteq \text {BPP}$. It is widely conjectured that $\text {NP} \not \subseteq \text {BPP}$ (see, e.g. Arora & Barak, 2009, Chapter 7). See the Appendix for more details.

13 There are no other constraints on *M* than specified in the definition of the problem Perfect-vs-Chance *M* can be thought of as a Turing machine. This is without loss of generality because, assuming the Invariance Thesis (van Emde Boas, 1990), a Turing machine can simulate any other computational machine with only polynomial-time overhead.

14 If *P* then *Q*. And $\lnot Q$. Therefore, $\lnot P$. Or, more formally, ($P \rightarrow Q, \lnot Q) \rightarrow \lnot P$.

15 Note that *A* is an algorithm that does not itself sample *s-b* pairs.

16 The selection of $A^{\star }$ is part of the internal workings of the proof. This does not commit to any particular interpretation of, e.g. what it means for an algorithm to be a good approximation.

17 Note that while our focus is on human-level or -like cognition, the intractability result has implications beyond this. It also applies to trying to use machine learning to reverse engineer systems that are at a similar *level* as human cognition, but possibly of a different *kind*, e.g. an octopus or a non-living domain-general computational system (i.e. the other meaning of AGI; see Table 1), because such domain-general systems meet the minimal computational assumptions as laid out in Section “Formalising AI-by-Engineering”.

18 Cf. ‘the Eliza effect’ (Weizenbaum, 1966; but see also Dillon, 2020). For instance, last year, Google fired Blake Lemoine, an engineer who believed that LaMDa (a large language model that he worked on) had become sentient (Fluckinger, 2022). It is wry that Lemoine was fired for expressing this (false) belief, while the Google CEO himself, Sundar Pichai, expressed no less unrealistic statements in a public interview (Pelley, 2023), suggesting that these systems have remarkable ‘emergent’ cognitive abilities, e.g. to know languages not trained on, being creative, able to reason and to plan (see Bender, 2023, for a critical analysis).

19 See for example the beginning of Newell (1970).

20 A few people seem to have recognized this, including Newell. He endorses the view that ‘the actual theories of cognitive psychology are to be expressed as artificial intelligence systems’ (p. 368) and goes on to observe that ‘[a]fter the fact, one can see that such a theory might have emerged within psychology (or linguistics) without the advent of the computer. In historical fact, the theory emerged by trying to program the computer to do non-numerical tasks and by trying to construct abstract theories of computation and logic’ (Newell, 1970, p. 373).

21 We acknowledge that computational modelling can also contribute to productive theory development without committing to computationalism (Guest & Martin, 2021; Morgan & Morrison, 1999). Here, we focus specifically on computationalist modelling, because we want to highlight that computationalism without makeism is possible. We also acknowledge existing critiques of ‘AI as computationalism’. Some of those critiques may target makeism (and/or dehumanisation; (Baria & Cross, 2021; van der Gun & Guest, 2023; Erscoi et al., 2023; Birhane & van Dijk, 2020; Birhane, 2021)) more than computationalism as a theoretical tool per se (but we will leave that judgement up to the critics). Be that as it may, we believe it is useful to explain how makeism and computationalism are dissociable, just as cognitivism and computationalism are dissociable (Villalobos & Dewhurst, 2017, 2018) and representationalism and computationalism are dissociable (Piccinini, 2008; Miłkowski, 2013, 2018).

22 For discussion of how possibly explanation, see, e.g. (Grüne-Yanoff, 2013; Bokulich, 2014, 2017; Sullivan, 2022).

23 Similarly, no finite set of ‘impressive’ observations about AIs/LLMs establishes that they exhibit a (human) cognitive capacity. This link can only be theoretically established; i.e., if we propose that a machine has a cognitive capacity *X*, we must also formally charactise *X* such that the machine’s capacity *Y* can be mathematically proven to be $X \equiv Y$ under relevant and naturalistic conditions, using appropriate proof techniques (see, e.g. Blokpoel & van Rooij, 2021, Chapter 5).

24 Tractable can be formalised, for instance, as needing a polynomial or fixed-parameter tractable amount of computational resources (van Rooij et al., 2019; van Rooij, 2008; Frixione, 2001).
